# Consistent MRI pattern in ADSS1 myopathy with variable clinical presentations: A Korean cohort study

**DOI:** 10.1371/journal.pone.0324116

**Published:** 2026-04-13

**Authors:** Soo-Hyun Kim, Yunjung Choi, Hyung Jun Park, Ji-Man Hong, Young-Chul Choi

**Affiliations:** 1 Department of Neurology, Gangnam Severance Hospital, Yonsei University College of Medicine, Seoul, Republic of Korea; 2 Rehabilitation Institute of Neuromuscular Disease, Gangnam Severance Hospital, Yonsei University College of Medicine, Seoul, Republic of Korea; 3 Department of Neurology, Yongin Severance Hospital, Yonsei University College of Medicine, Yongin, Gyeonggi-do, Republic of Korea; 4 Department of Neurology, SCL Hanaro Leaders Clinic, Seoul, Republic of Korea; University of Miami Miller School of Medicine: University of Miami School of Medicine, UNITED STATES OF AMERICA

## Abstract

ADSS1 myopathy (previously referred to as ADSSL1 myopathy) is a rare autosomal recessive muscle disease caused by mutations in the ADSS1 gene, which encodes an enzyme critical for purine nucleotide synthesis. First characterized in Korean patients in 2016, the disease exhibits phenotypic variability in its clinical presentation. We conducted a retrospective cohort study of 30 patients with genetically confirmed ADSS1 myopathy (18 males and 12 females) at Gangnam Severance Hospital from 2002 to 2024. The patients were classified into proximal-onset (n = 9) and distal-onset (n = 20) groups based on the location of initial muscle weakness, with one patient presenting with isolated hyperCKemia. Clinical assessments, genetic analyses, and muscle MRI were performed on 10 patients to evaluate clinical-radiological correlations. The median age at symptom onset was 8.0 years [Interquartile range (IQR): 7.0–14.0] with a median disease duration of 24.0 years [IQR: 17.0–34.0]. The most common initial symptoms were slow running (66.7%), early fatigue (16.7%), and gait disturbances (10.0%). Facial involvement was observed in 80.0% of the patients and oropharyngeal dysfunction in 56.7%. The median serum creatine kinase level was 214.0 IU/L [IQR: 125.0–394.0]. Genetic analysis revealed five pathogenic ADSS1 variants, with c.781G > A (51.7% of alleles) and c.919del (40.0% of alleles) being the most prevalent. Most patients (73.3%) were compound heterozygous for the two variants. Despite the clinical heterogeneity between the proximal- and distal-onset groups, none of the clinical differences were statistically significant. Muscle MRI revealed a remarkably consistent pattern of preferential involvement of the distal lower limb muscles, particularly the gastrocnemius and soleus muscles, regardless of the initial clinical presentation. This study, which represents the largest Korean ADSS1 myopathy cohort to date, highlights the striking discordance between clinical phenotypes and radiological findings. Although the clinical presentations varied considerably, MRI revealed consistent distal dominant muscle involvement patterns across all patients. This suggests that the underlying pathological process follows a predictable anatomical distribution independent of the initial symptomatic muscle groups. Our findings support the utility of muscle MRI as a valuable diagnostic tool for ADSS1 myopathy and suggest its conceptualization as a unified disease entity with a common pathophysiological mechanism involving selective muscle vulnerability based on metabolic requirements.

## Introduction

Adenylosuccinate synthase 1 (ADSS1) myopathy is a rare autosomal recessive muscle disease caused by mutations in the *ADSS1*. The causative gene for this myopathy was previously referred to as *ADSSL1* but has been renamed *ADSS1* according to the updated gene nomenclature.[1] The *ADSS1* encodes adenylosuccinate synthetase 1, an enzyme critical for the purine nucleotide synthesis pathway [2,3], and its dysfunction is thought to impair energy homeostasis in muscle cells, leading to progressive muscular dysfunction [4–6]. First characterized in Korean patients in 2016 [2], ADSS1 myopathy has since been identified in other populations including Japan, India, Europe, China, and the United States [7–11]. To date, over a hundred genetically confirmed cases have been reported worldwide, establishing ADSS1 myopathy as a distinct hereditary myopathy [10].

Initial reports described ADSS1 myopathy as a childhood-onset, slowly progressive, distal myopathy with facial and oropharyngeal involvement [2,3]. Subsequent studies revealed wider phenotypic variability, with both proximal and distal onset presentations and varying degrees of severity across different ethnic groups [7,8,10,12]. Notably, the founder mutation c.781G > A (p.Asp261Asn) has been frequently reported in East Asian populations, suggesting a regional genetic signature with potential implications for diagnosis and screening [3,8,10]. Radiological studies have demonstrated distinctive patterns of muscle involvement predominantly affecting the distal muscles [13,14]. Recent studies have progressively expanded our understanding of the clinical spectrum of the disease, highlighting ADSS1 myopathy as an emerging entity of global significance despite its initial characterization in Korean patients [7–10].

Despite these advances, significant questions remain unresolved regarding ADSS1 myopathy. Current phenotypic classifications are inadequate for capturing the full clinical spectrum, and genotype-phenotype correlations remain unclear [6,10]. Moreover, significant variability in disease severity among patients with identical genotypes suggests the presence of unidentified modifying factors [7,8,10,12]. In other neuromuscular disorders, such as dysferlinopathy, muscle MRI has proven invaluable in redefining clinical subtypes and identifying shared pathological signatures [14–17]. Although ADSS1 myopathy is associated with distinctive progressive proximal and distal muscle weakness [2,3,8], systematic imaging studies remain scarce. In landmark studies of other myopathies, distinct phenotypes converged on common MRI patterns, supporting the conceptual unification of these disorders [15,18]. This suggests that MRI can serve as a powerful tool for phenotype refinement and disease monitoring beyond clinical observation alone [13,14].

To date, however, no study has quantitatively characterized the MRI patterns of ADSS1 myopathy. Our study provides the first systematic radiological evaluation to define its disease-specific MRI signature and to clarify the clinical-radiological relationship. By classifying patients according to the anatomical location of symptom onset (proximal vs. distal) and correlating these classifications with muscle imaging findings and genetic profiles, we assessed whether the current clinical subtypes reflected the underlying pathophysiology.

## Methods

### Patient selection and genetic analysis

A retrospective cohort study was conducted on 30 patients diagnosed with ADSS1 myopathy at the Gangnam Severance Hospital between August 2002 and April 2024. Diagnosis was established based on clinical presentation, muscle biopsy findings when available, and genetic confirmation of pathogenic variants of ADSS1. Genetic confirmation was performed using targeted next-generation sequencing (NGS), which included a custom panel designed for neuromuscular disorders incorporating the ADSS1. Sanger sequencing was subsequently performed on a subset of patients to confirm the variants identified by NGS. The variants were interpreted according to the 2015 American College of Medical Genetics and Genomics and the Association for Molecular Pathology (ACMG-AMP) guidelines and classified as pathogenic.

### Clinical evaluation and phenotypic classification

Comprehensive clinical data, including demographic information (sex and age), age at onset, initial presenting symptoms, and disease progression patterns, were collected and analyzed. We classified the patients into two distinct phenotypic groups based on the anatomical location of their initial weakness at symptom onset: proximal-onset type, defined as initial weakness predominantly affecting the shoulder and/or pelvic girdle muscles, and distal-onset type, defined as initial weakness predominantly affecting distal limb muscles such as the hand, forearm, ankle, and/or foot muscles. Patients were classified into proximal-onset and distal-onset groups according to the anatomical location of the initial muscle weakness. Neurological examination performed around the time of MRI acquisition included Medical Research Council (MRC) based manual muscle testing of proximal and distal muscle groups, allowing comparison between clinical classification and imaging findings.

Detailed physical and neurological examinations were performed on all patients. Manual muscle testing (MMT) using the Medical Research Council (MRC) scale was conducted to assess muscle strength in both the proximal (shoulder abductors, elbow flexors/extensors, hip flexors/extensors, knee flexors/extensors) and distal muscle groups (wrist flexors/extensors, finger flexors/extensors, ankle dorsiflexors/plantar flexors). Functional status was evaluated based on ambulatory capacity, classifying patients as independently ambulating, requiring assistive devices, or non-ambulatory.

Laboratory investigations included measurement of serum creatine kinase (CK) levels in 29 of 30 patients. When clinically indicated, cardiological evaluations (electrocardiography and echocardiography) and respiratory function tests (spirometry with forced vital capacity) were performed.

### Muscle MRI analysis using mercuri’s scale

Muscle imaging studies were performed using magnetic resonance imaging (MRI) to evaluate the patterns of muscle involvement in 10 patients (2 proximal-onset, 8 distal-onset).

MRI was performed using a 3.0-T MAGNETOM Vida scanner (Siemens Healthineers, Erlangen, Germany). Axial T1-weighted spin-echo images were acquired from the pelvis to the lower legs (TR/TE = 655/9 ms, slice thickness = 6 mm, gap = 7.2 mm, FOV = 500 × 344 mm, matrix = 512 × 246).

All images were visually assessed and classified using Mercuri’s scale, which grades the severity of muscle involvement from 0 to 4 based on the degree of fatty infiltration as follows: grade 0 (normal appearance); grade 1 (occasional small areas of hyperintensity); grade 2 (fatty replacement in less than 30% of the muscle); grade 3 (fatty replacement in 30–60% of the muscle); and grade 4 (fatty replacement in more than 60% of the muscle) [13]. Any discrepancies were resolved by consensus. All MRI scans underwent independent evaluation by two neurologists who were blinded to clinical data. Inter-rater agreement was assessed at the grade level, with all discrepancies limited to a maximum of one grade difference. In cases where a one-grade discrepancy was observed, the images were collaboratively re-examined by both reviewers to reach a consensus score. Final grades were determined thorough this consensus process without any averaging or rounding of individual scores.

### Statistical analysis

Clinical, genetic, and imaging data were analyzed using descriptive statistics and comparative analyses. Continuous variables were expressed as means ± standard deviation (SD) or medians with interquartile ranges (IQR), while categorical variables were presented as frequencies and percentages. Comparisons between the proximal-onset and distal-onset groups were performed using Fisher#39;s exact test for categorical variables and the Mann-Whitney U test for continuous variables. Statistical significance was set at p < 0.05. Statistical analyses were performed using SPSS Statistics, version 27 (IBM Corp., Armonk, NY, USA).

### Ethical approval and consent

This study was approved by the Institutional Review Board (IRB) of Gangnam Severance Hospital (IRB number: 3-2025-0022). Written informed consent was obtained from all participants or their legal guardians for the collection and analysis of clinical data, genetic testing, and when applicable, muscle biopsy specimens. This study was conducted in accordance with the principles of the Declaration of Helsinki.

## Results

### Demographic and clinical characteristics (Table 1 and S1 Table)

Our cohort consisted of 30 Korean patients with genetically confirmed ADSS1 myopathy (18 males, 12 females). The median age at symptom onset was 8.0 years [interquartile range (IQR): 7.0–14.0], with patients examined at a median age of 38.0 years [IQR: 25.0–49.8], representing a median disease duration of 24.0 years [IQR: 17.0–34.0], as summarized in Table 1. Based on the anatomical location of the initial weakness, the patients were classified into the proximal-onset (n = 9) and distal-onset (n = 20) groups, with one patient presenting with isolated hyperCKemia without muscle weakness.

**Table 1:** Clinical characteristics of 30 patients with ADSS1 myopathy, including a comparison between proximal and distal onset groups (n (%) or median [interquartile range]). Detailed information for the 10 MRI-evaluated patients is provided in S2 and S3 Table.

Characteristic	Total (n = 30)	Proximal-onset (n = 9)	Distal-onset (n = 20)	*p-value
**Male: Female**	18:12	7:2	10:10	
**Age of symptom onset, years**	8.0 [7.0–14.0]	8.0 [7.0-14.0]	8.0 [6.8-13.1]	0.793
**Age of examination, years**	38.0 [25.0–49.8]	39.0 [24.0-43.0]	38.0 [28.0-55.3]	0.324
**Disease duration, years**	24.0 [17.0–34.0]	23.0 [16.0-34.0]	25.5 [17.0-40.3]	0.257
**Muscle MRI performed, n**	10	2	8	
**Initial clinical symptoms**				
Slow runner	20 (66.7)	5 (55.6)	15 (75.0)	0.396
Gait disturbance	3 (10.0)	0 (0)	3 (15.0)	0.532
Early fatigue	5 (16.7)	3 (33.3)	2 (10.0)	0.287
Stair climbing difficulty	3 (10.0)	1 (11.1)	2 (10.0)	1.000
Myalgia and hyperCKemia	1 (3.3)	0 (0)	0 (0)	1.000
**Associated symptoms (%)**				
Respiratory insufficiency	6 (20.0)	2 (22.2)	4 (20.0)	1.000
Cardiac impairment	3 (10.0)	1 (11.1)	2 (10.0)	1.000
Intellectual disability	0 (0)	0 (0)	0 (0)	1.000
**Other motor symptoms (%)**				
Facial diplegia	24 (80.0)	8 (88.9)	16 (80.0)	1.000
Oropharyngeal dysfunction	17 (56.7)	5 (55.6)	12 (60.0)	1.000
Dysarthria	13 (43.3)	5 (55.6)	8 (40.0)	0.688
**Serum level of CK, IU/L**	214.0 [125.0-394.0]	247.0 [182.0-323.0]	178.0 [98.0-417.5]	0.923
**Serum level of Uric acid, mg/dL**	5.6 [4.7-6.8]	6.1 [5.6-6.7]	5.1 [4.4-7.1]	0.640

CK, creatine kinase

* P-values indicate the level of significance in the comparison between the proximal and distal onset groups.

The most common initial symptoms were slow running (66.7%), early fatigue (16.7%), gait disturbance (10.0%), and difficulty climbing stairs (10.0%). On examination, most patients (66.7%) exhibited greater weakness in the distal muscles than in the proximal muscles. Notably, facial muscle involvement (facial diplegia) was observed in 80.0% of patients. Laboratory findings revealed a median serum creatine kinase (CK) level of 214.0 IU/L [IQR: 125.0–394.0] and a median uric acid level of 5.6 mg/dL [IQR: 4.7–6.8]. There were no statistically significant differences between proximal- and distal-onset groups in age at onset, CK, urate, or disease duration (all p > 0.05). Other systemic or cranio-bulbar features (facial weakness, dysarthria, respiratory or cardiac involvement) were uncommon and are summarized in S1 Table.

### Genetic analysis (Fig 1–2, and Table 2)

**Fig 1:**
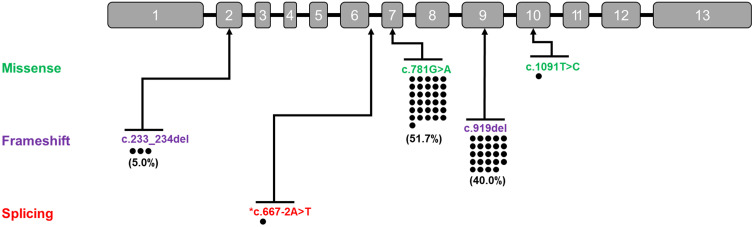
Pathogenic variants in the *ADSS1* gene identified in Korean patients with ADSS1 myopathy. Schematic representation of the *ADSS1* gene showing the distribution and frequency of the five identified variants. Numbered boxes represent exons [1–13]. Dots represent individual alleles from 30 patients (60 alleles total), with variants color-coded by mutation type: missense (green), frameshift (purple), and splicing (red). The most common variants were **c.**781G > A (51.7%) and c.919del (40.0%). Asterisk indicates the novel splice-site variant c.667-2A > T.

Genetic analysis of 30 patients (60 alleles) revealed five distinct pathogenic variants of ADSS1 (Fig 1).

**Table 2:** Genotype distribution in 30 Korean patients with ADSS1 myopathy.

Zygosity of ADSS1 myopathy patients		Number/total number of variants (%)
c.781G > A + c.919del	Heterozygote	22/30 (73.3%)
c.781G > A	Homozygote	2/30 (6.7%)
c.781G > A + c.233_234del	Heterozygote	3/30 (10.0%)
c.919del	Homozygote	1/30 (3.3%)
c.781G > A + c.667-2A > T	Heterozygote	1/30 (3.3%)
c.781G > A + c.1091T > C	Heterozygote	1/30 (3.3%)

All variants were classified as pathogenic according to the 2015 the 2015 American College of Medical Genetics and Genomics and the Association for Molecular Pathology (ACMG–AMP) guidelines.

**Fig 2:**
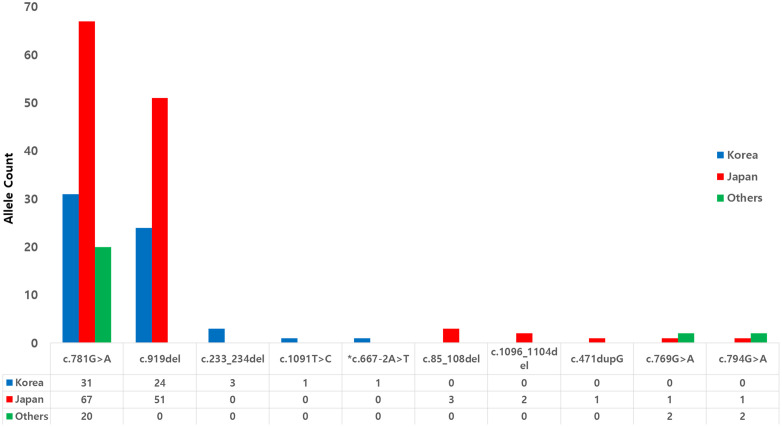
Comparison of ADSS1 variant distributions across different populations. Bar graph showing the allele counts of ADSS1 pathogenic variants in Korean (blue), Japanese (red), and other populations (green). Note the predominance of **c.**781G > A and c.919del variants in both Korean and Japanese populations. Greater variation is observed in other populations(India, Turkey, and USA) with variants not detected in East Asian patients.

The most prevalent variant was c.781G > A (p.Asp261Asn), which accounted for 51.7% (31/60) of all alleles, followed by c.919del (p.Ile307Serfs25), which accounted for 40.0% (24/60). The less common variants included c.233_234del (p.Lys78Argfs33) at 5.0% (3/60), c.1091T > C (p.Leu364Pro) at 1.7% (1/60), and a novel splice-site variant c.667-2A > T (p.?) also at 1.7% (1/60). Zygosity analysis revealed that most patients (73.3%, 22/30) were compound heterozygotes for c.781G > A and c.919del (Table 2). Other genotypes included homozygosity of c.781G > A (6.7%, 2/30), compound heterozygosity of c.781G > A and c.233_234del (10.0%, 3/30), homozygosity of c.919del (3.3%, 1/30), and compound heterozygosity of c.781G > A with either c.667-2A > T or c.1091T > C (3.3% each, 1/30 each). All variants except c.667-2A > T have been previously reported.

An international comparison of variant distributions demonstrated similarities between Korean and Japanese patients, with c.781G > A and c.919del as the predominant variants in both populations (Figure 2).

In our Korean cohort, these two variants accounted for more than 90% of all disease alleles. In contrast, patients from other countries showed different variant patterns with additional variants (c.769G > A and c.794G > A) that were not observed in our Korean cohort. The Japanese cohort exhibited the highest numbers of c.781G > A and c.919del alleles among the reported populations. The c.667-2A > T variant identified in one patient represents a previously unreported splice-site variation in ADSS1, affecting the canonical acceptor splice site of intron 6.

### Muscle MRI findings (Figs 3,4)

**Fig 3:**
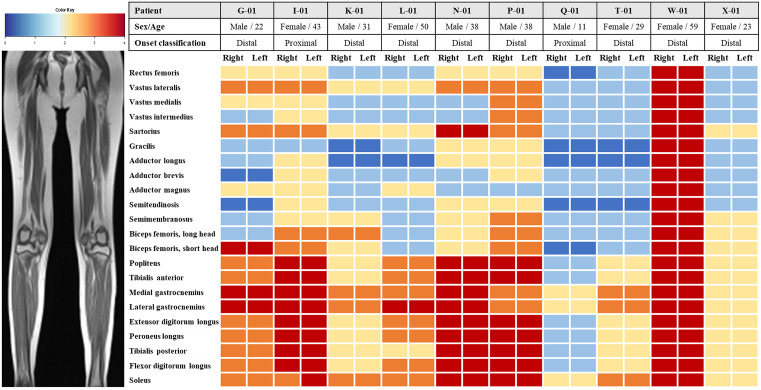
Fatty infiltration pattern in lower limb muscles of ADSS1 myopathy patients assessed using T1-weighted MRI. Heat map displaying the severity of fatty infiltration in 22 individual muscles of both lower limbs from 10 patients with ADSS1 myopathy, graded according to Mercuri#39;s scale from 0 (blue, normal) to 4 (red, severe fatty replacement). Patients are arranged in data collection order (not ordered by total Mercuri score) and grouped by their clinical onset classification. Note the consistent pattern of predominant involvement of distal muscles, particularly gastrocnemius and soleus, across all tested patients. Left and right sides are displayed separately for completeness, although asymmetry was rare (observed in one patient).

Muscle MRI examinations were performed in 10 patients (two with proximal onset and eight with distal onset) to evaluate the patterns of muscle involvement. Neurological examinations performed around the time of MRI confirmed that the distribution of muscle weakness at imaging was consistent with the clinical phenotype, allowing direct comparison between strength patterns and imaging findings. The severity of fatty infiltration in the lower limb muscles was assessed using the Mercuri scale and visualized as a heat map (Fig 3). Heat map analysis (Fig 3) demonstrated that the most severely affected muscles were predominantly located in the distal compartments of the lower limbs.

**Fig 4:**
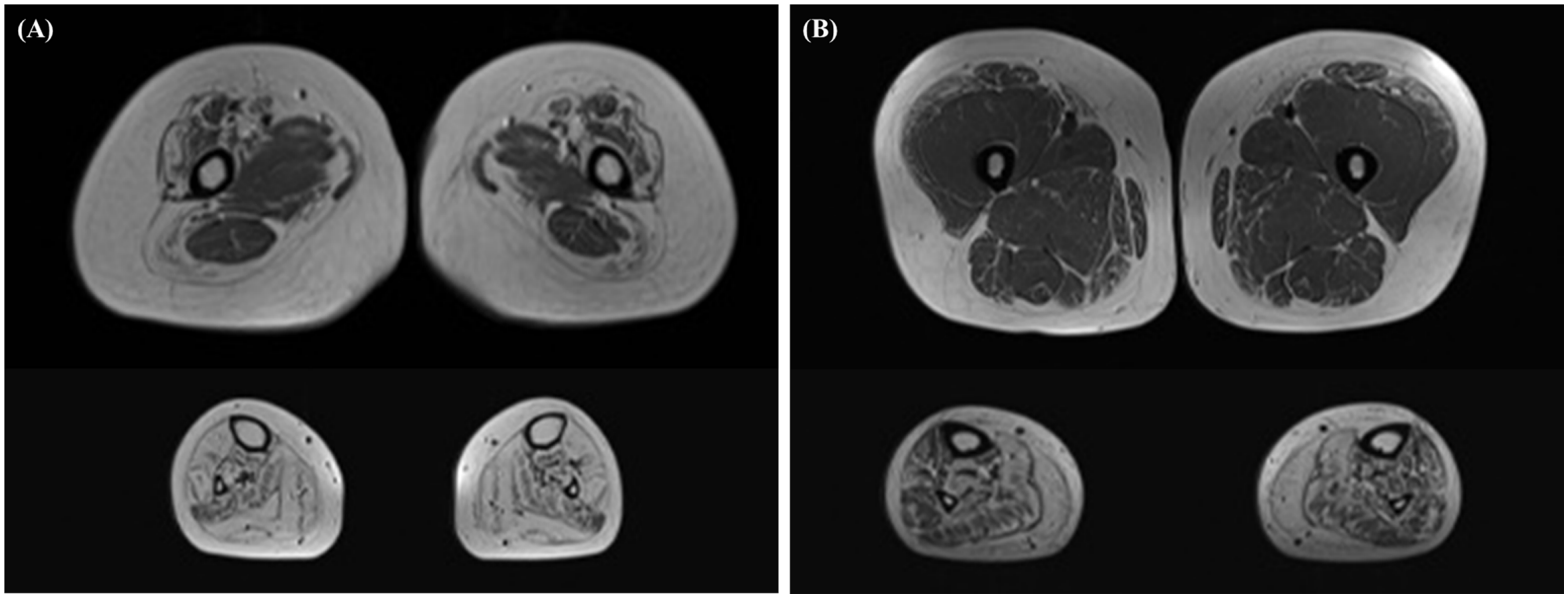
Representative T1-weighted MRI images from proximal-onset and distal-onset ADSS1 myopathy patients. Axial T1-weighted MRI sections at thigh (upper panels) and calf (lower panels) levels from (A) a proximal-onset patient (G-01) and (B) a distal-onset patient (L-01). Both patients show similar patterns of muscle involvement with predominant fatty replacement in distal muscles, particularly the gastrocnemius and soleus, while proximal muscles are relatively less affected.

Specifically, the medial and lateral gastrocnemius muscles showed the most extensive fatty replacement (grades 3−4) in the majority of patients, followed by the soleus muscle. The tibialis anterior, popliteus, extensor digitorum longus, peroneus longus, and tibialis posterior muscles also showed moderate-to-severe involvement. In contrast, the proximal muscles generally displayed less severe fatty infiltration, except for the vastus lateralis and sartorius, which were more frequently and severely affected than other proximal muscle groups. Representative MRI images from a proximal-onset patient (G-01) and a distal-onset patient (L-01) are shown in Fig 4.

Both patients exhibited predominant fatty replacement in the distal lower limb muscles, particularly in the posterior compartment, whereas the proximal muscles were relatively spared.

## Discussion

This study represents the largest single-center cohort analysis of ADSS1 myopathy in Korea and provides comprehensive insights into its clinical, genetic, and radiological features. Our findings highlight a striking contrast between the clinical heterogeneity of the initial presentations and the remarkably consistent pattern of muscle involvement observed in imaging studies. One of the most significant findings of our study was the discordance between clinical phenotypes and radiological manifestations of ADSS1 myopathy. Clinically, we observed considerable heterogeneity in the anatomical sites of the initial symptoms, allowing us to classify the patients into proximal-onset (n = 9) and distal-onset (n = 20) groups. These groups exhibited differences in their clinical presentations. Patients with proximal onset exhibited higher rates of early fatigue and facial weakness, whereas those with distal onset more frequently presented with slow running and gait disturbances. However, none of these clinical distinctions were statistically significant, suggesting a substantial overlap between the phenotypic subgroups. This clinical variability is consistent with the results of previous reports. While ADSS1 myopathy was initially described as a predominantly distal myopathy in Korean patients [2,3], the phenotypic spectrum has since expanded. Recent studies have documented both distal and proximal muscle weakness in non-Korean patients and variable muscle symptoms affecting the proximal and/or distal leg muscles, tongue, masseter, diaphragm, and paraspinal muscles in Japanese cohorts [7,8]. Additional clinical features, including significant seasonal fluctuations and decremental responses to repetitive nerve stimulation, have been reported in patients from other populations [12]. In contrast to this clinical variability, muscle MRI revealed a remarkably uniform pattern of muscle involvement across all examined patients, regardless of their initial clinical presentation. Both proximal- and distal-onset patients demonstrated predominant fatty replacement in the distal lower limb muscles, particularly the gastrocnemius and soleus, with relative sparing of the proximal muscles (except for the vastus lateralis and sartorius, which showed earlier involvement than the other proximal muscles). This consistent distal-dominant pattern on MRI suggests that the underlying pathological process follows a predictable anatomical distribution, independent of the initial symptomatic muscle groups. This discrepancy between clinical presentation and imaging findings raises fundamental questions regarding the pathophysiology of ADSS1 myopathy. A comparable clinical–radiological dissociation has been reported in dysferlinopathy, where patients classified by initial presentation as either limb-girdle (LGMD2B) or distal onset (Miyoshi myopathy) exhibit identical MRI patterns of muscle involvement (Paradas et al., Neurology, 2010). In that study, as in ours, classification was based on the initial site of weakness, as standardized strength scores concurrent with imaging were not uniformly available. Nevertheless, the MRI findings revealed a consistent underlying pattern that redefined the disease as a single pathological spectrum. Similarly, the present study demonstrates that despite variable clinical onsets, ADSS1 myopathy follows a uniform distal-dominant MRI distribution, supporting its conceptualization as a unified disorder rather than distinct proximal and distal forms. This suggests that factors beyond the underlying pattern of muscle involvement, such as individual compensatory mechanisms, daily activity patterns, or genetic modifiers, may influence the muscle groups that first manifest clinically detectable weakness. However, consistent MRI patterns may reflect the true biological vulnerability of specific muscle groups to ADSS1 deficiency [8,14]. Our findings strongly support the utility of muscle MRI as a valuable diagnostic tool for ADSS1 myopathy. The characteristic pattern of distal dominant muscle involvement, particularly the gastrocnemius, soleus, and tibialis anterior muscles, appears to be consistent with the radiological signature of this disorder. This pattern was observed in all examined patients, suggesting high sensitivity for detecting ADSS1 myopathy. This radiological signature may be particularly valuable in diagnostically challenging cases, such as those presenting with isolated facial weakness or minimal limb symptoms, where the characteristic distal-dominant MRI changes help confirm the diagnosis, especially when initial genetic testing is inconclusive. In these scenarios, recognition of the characteristic MRI pattern can guide targeted genetic analysis and potentially expedite diagnosis [13,14]. Furthermore, in the context of the East Asian founder mutations identified in our cohort (c.781G > A and c.919del), the combination of this distinctive MRI pattern with targeted genetic testing for these common variants may represent an effective diagnostic algorithm for suspected ADSS1 myopathy in Korean patients. This observation parallels findings in other neuromuscular disorders, where muscle MRI has significantly contributed to phenotype refinement and diagnostic accuracy. Previous studies have demonstrated distinct muscle imaging patterns in various myopathies that help separate genetically heterogeneous subtypes and reveal specific patterns that aid in diagnosis [16,18]. Notably, muscle MRI has been used to redefine the phenotypes of other neuromuscular disorders, demonstrating that patients with clinically distinct presentations may show identical patterns of muscle involvement in imaging [15]. Beyond its diagnostic utility, muscle MRI provides unique insights into the natural history and pathophysiology of ADSS1 myopathy, which cannot be obtained through clinical examination alone. The ability to visualize and quantify the extent of muscle involvement across different muscle groups allows for a more precise assessment of disease severity and progression than is possible with clinical strength testing, particularly in muscles that are difficult to isolate on examination [13]. Despite clinical heterogeneity, the consistent distal dominant pattern observed on MRI supports the conceptualization of ADSS1 myopathy as a unified disease entity with a common underlying pathophysiological mechanism. This perspective parallels the evolution of our understanding of other neuromuscular disorders, in which initially distinct clinical syndromes were later recognized as different manifestations of the same underlying pathological process [15]. The preferential involvement of distal muscles, particularly those with high energy demands such as the gastrocnemius and soleus, may reflect their heightened vulnerability to disruptions in energy metabolism caused by ADSS1 deficiency [4,19]. These muscles, which are continuously engaged in postural maintenance and locomotion, may be particularly dependent on efficient purine nucleotide cycling and ATP regeneration [4,5]. The purine nucleotide cycle, comprising adenylosuccinate synthetase (ADSS1), adenylosuccinate lyase, and adenosine monophosphate (AMP) deaminase, plays a crucial role in muscle energy metabolism during intense exercise, contributing to ATP regeneration and providing Krebs cycle intermediates [4]. The selective vulnerability of specific proximal muscles (vastus lateralis and sartorius) can be explained by their metabolic profiles or fiber-type composition, which may render them more susceptible to the metabolic consequences of ADSS1 dysfunction than other proximal muscles. This hypothesis is supported by transcriptome analysis demonstrating that genes involved in purine nucleotide cycling (ADSS1, ADSL, and AMPD1) are significantly downregulated in the muscle tissues of patients with ADSS1 myopathy [6]. This unified model of ADSS1 myopathy, which is characterized by selective muscle vulnerability based on metabolic requirements rather than anatomical location, has important implications for understanding disease progression and developing therapeutic strategies. This suggests that treatments targeting the fundamental metabolic disruption caused by ADSS1 deficiency may be effective regardless of the clinical phenotype.

Our study has several limitations. First, despite being the largest Korean cohort with ADSS1 myopathy to date, our sample size was relatively small, particularly for subgroup analyses. Second, muscle MRI was performed in only a subset of the patients (10 of 30), potentially limiting the generalizability of our radiological findings. Third, the cross-sectional nature of our imaging data precludes definitive conclusions regarding the temporal progression of muscle involvement. Additionally, quantitative MRI fat-fraction or biochemical correlations and RNA-based analyses were not performed due to lack of available data and IRB restrictions. Future research should address these limitations through longitudinal studies that track both clinical progression and changes in muscle MRI findings over time. It is particularly important to investigate the temporal relationship between the initial onset of clinical symptoms and the development of characteristic MRI patterns. Such studies could clarify whether radiological changes precede clinical manifestations, potentially identifying the pre-symptomatic phase during which therapeutic interventions might be most effective. Additionally, correlation studies between muscle MRI findings and functional measures of ADSS1 enzyme activity or markers of purine metabolism could provide deeper insights into the pathophysiological mechanisms underlying the selective muscle vulnerability observed in this disorder [6,20]. Recent pilot investigations using electrical impedance myography have demonstrated the potential for noninvasive characterization of muscle integrity in patients with ADSS1 myopathy and could complement MRI findings in future studies [21]. Further exploration of the potential genetic modifiers influencing the clinical manifestations of ADSS1 myopathy in the setting of identical pathogenic variants would also be valuable [10].

## Supporting information

S1 Table Clinical and laboratory features of 30 patients with ADSS1 myopathy. The table summarizes demographic, clinical, and laboratory data of all genetically confirmed patients. Onset classification was based on the initial muscle weakness pattern (proximal or distal). Variants are described according to the NM_199165 transcript.
(XLSX)

S2 Table Clinical and MRI-related variables for 10 ADSS1 myopathy patients undergoing muscle MRI. Onset type was defined by the initial weakness pattern. MRC scores were obtained around the time of MRI, and disease duration was calculated as the interval between onset and MRI.
(XLSX)

S3 Table Individual Mercuri grades (0–4) for 22 lower-limb muscles in 10 ADSS1 myopathy patients. Left (L) and right (R) values are presented under each patient ID to correspond directly with figure 3. All patients showed symmetrical involvement except for one (I-01), who exhibited mild right-dominant asymmetry in the posterior calf muscles.
(XLSX)
