# Correction: Consistent MRI pattern in ADSS1 myopathy with variable clinical presentations: A Korean cohort study

**DOI:** 10.1371/journal.pone.0351854

**Published:** 2026-06-16

**Authors:** Soo-Hyun Kim, Yunjung Choi, Hyung Jun Park, Ji-Man Hong, Young-Chul Choi

An additional affiliation is missing for the first author. Soo-Hyun Kim is also affiliated with the Ewha Womans University College of Medicine, Seoul, Korea.
